# Supplementary material for: Multi‐dataset identification of innovative feature genes and molecular mechanisms in keratoconus
Source: J Cell Mol Med. 2024 Sep 19;28(18):e70079. doi: 10.1111/jcmm.70079 (PMC11412914; doi:10.1111/jcmm.70079)
Supplement: Supplementary file 2 — Material S2. [file JCMM-28-e70079-s002.zip › 7.GSEA analysis/4.MYRF/KEGG_TGF_BETA_SIGNALING_PATHWAY.html]

Details for gene set KEGG\_TGF\_BETA\_SIGNALING\_PATHWAY[GSEA]

|  || Dataset | MYRF.group.cls#h\_versus\_l |
| Phenotype | group.cls#h\_versus\_l |
| Upregulated in class | l |
| GeneSet | KEGG\_TGF\_BETA\_SIGNALING\_PATHWAY |
| Enrichment Score (ES) | -0.48614332 |
| Normalized Enrichment Score (NES) | -1.640796 |
| Nominal p-value | 0.004 |
| FDR q-value | 0.25299612 |
| FWER p-Value | 0.523 |
Table: GSEA Results Summary

  

Fig 1: Enrichment plot: KEGG\_TGF\_BETA\_SIGNALING\_PATHWAY      
 Profile of the Running ES Score & Positions of GeneSet Members on the Rank Ordered List

  

| SYMBOL | TITLE | RANK IN GENE LIST | RANK METRIC SCORE | RUNNING ES | CORE ENRICHMENT || 1 | GDF6 | na | 1823 | 0.372 | -0.0288 | No |
| 2 | AMHR2 | na | 2900 | 0.300 | -0.0406 | No |
| 3 | NODAL | na | 3199 | 0.286 | -0.0309 | No |
| 4 | NOG | na | 3260 | 0.282 | -0.0145 | No |
| 5 | GDF7 | na | 4744 | 0.230 | -0.0425 | No |
| 6 | BMP8B | na | 4981 | 0.223 | -0.0350 | No |
| 7 | ACVR2B | na | 5540 | 0.206 | -0.0379 | No |
| 8 | ZFYVE9 | na | 6291 | 0.183 | -0.0478 | No |
| 9 | BMPR1B | na | 7766 | 0.144 | -0.0811 | No |
| 10 | SMAD5 | na | 8694 | 0.123 | -0.1000 | No |
| 11 | ROCK1 | na | 8751 | 0.121 | -0.0938 | No |
| 12 | ZFYVE16 | na | 9229 | 0.111 | -0.1005 | No |
| 13 | CREBBP | na | 9688 | 0.102 | -0.1071 | No |
| 14 | ACVR1C | na | 9724 | 0.101 | -0.1017 | No |
| 15 | BMPR2 | na | 10193 | 0.093 | -0.1092 | No |
| 16 | CHRD | na | 10558 | 0.086 | -0.1142 | No |
| 17 | LEFTY1 | na | 10844 | 0.081 | -0.1173 | No |
| 18 | RPS6KB2 | na | 11604 | 0.069 | -0.1348 | No |
| 19 | BMP8A | na | 12293 | 0.059 | -0.1508 | No |
| 20 | ACVRL1 | na | 12475 | 0.057 | -0.1524 | No |
| 21 | SP1 | na | 13004 | 0.050 | -0.1644 | No |
| 22 | INHBC | na | 13034 | 0.050 | -0.1621 | No |
| 23 | SMAD4 | na | 13356 | 0.045 | -0.1684 | No |
| 24 | ROCK2 | na | 13790 | 0.040 | -0.1783 | No |
| 25 | RPS6KB1 | na | 13890 | 0.039 | -0.1787 | No |
| 26 | MAPK3 | na | 14382 | 0.034 | -0.1907 | No |
| 27 | RBX1 | na | 14789 | 0.030 | -0.2005 | No |
| 28 | PPP2R1B | na | 15086 | 0.027 | -0.2073 | No |
| 29 | SMAD1 | na | 16667 | 0.011 | -0.2522 | No |
| 30 | SKP1 | na | 17295 | 0.006 | -0.2699 | No |
| 31 | TFDP1 | na | 17568 | 0.004 | -0.2775 | No |
| 32 | SMAD3 | na | 17747 | 0.002 | -0.2825 | No |
| 33 | RBL1 | na | 17757 | 0.002 | -0.2827 | No |
| 34 | RBL2 | na | 18064 | -0.001 | -0.2914 | No |
| 35 | SMAD2 | na | 18091 | -0.001 | -0.2921 | No |
| 36 | RHOA | na | 18827 | -0.008 | -0.3128 | No |
| 37 | LTBP1 | na | 20162 | -0.024 | -0.3498 | No |
| 38 | MAPK1 | na | 20406 | -0.027 | -0.3551 | No |
| 39 | BMPR1A | na | 21219 | -0.036 | -0.3762 | No |
| 40 | ID1 | na | 21235 | -0.036 | -0.3743 | No |
| 41 | CUL1 | na | 21468 | -0.039 | -0.3785 | No |
| 42 | CDKN2B | na | 21823 | -0.044 | -0.3859 | No |
| 43 | ACVR2A | na | 21886 | -0.044 | -0.3849 | No |
| 44 | TGFB2 | na | 22177 | -0.048 | -0.3902 | No |
| 45 | THBS1 | na | 22189 | -0.048 | -0.3874 | No |
| 46 | BMP7 | na | 22323 | -0.050 | -0.3880 | No |
| 47 | SMURF2 | na | 22496 | -0.052 | -0.3897 | No |
| 48 | PPP2R1A | na | 22646 | -0.054 | -0.3905 | No |
| 49 | THBS3 | na | 24014 | -0.076 | -0.4250 | No |
| 50 | E2F5 | na | 24596 | -0.086 | -0.4363 | No |
| 51 | LEFTY2 | na | 24899 | -0.091 | -0.4392 | No |
| 52 | TGFBR2 | na | 24993 | -0.093 | -0.4359 | No |
| 53 | TGFBR1 | na | 25528 | -0.103 | -0.4447 | No |
| 54 | PPP2CB | na | 25840 | -0.109 | -0.4467 | No |
| 55 | THBS4 | na | 26080 | -0.115 | -0.4462 | No |
| 56 | SMURF1 | na | 26375 | -0.121 | -0.4469 | No |
| 57 | THBS2 | na | 26821 | -0.131 | -0.4514 | No |
| 58 | PPP2CA | na | 26826 | -0.131 | -0.4431 | No |
| 59 | EP300 | na | 27396 | -0.145 | -0.4502 | No |
| 60 | BMP6 | na | 27515 | -0.148 | -0.4441 | No |
| 61 | PITX2 | na | 27573 | -0.150 | -0.4362 | No |
| 62 | MYC | na | 27707 | -0.153 | -0.4302 | No |
| 63 | ACVR1 | na | 29069 | -0.193 | -0.4571 | No |
| 64 | BMP5 | na | 29906 | -0.220 | -0.4671 | Yes |
| 65 | AMH | na | 29982 | -0.223 | -0.4550 | Yes |
| 66 | E2F4 | na | 30051 | -0.225 | -0.4425 | Yes |
| 67 | ID3 | na | 30986 | -0.263 | -0.4526 | Yes |
| 68 | GDF5 | na | 32150 | -0.327 | -0.4652 | Yes |
| 69 | SMAD6 | na | 32211 | -0.330 | -0.4457 | Yes |
| 70 | INHBB | na | 32328 | -0.338 | -0.4274 | Yes |
| 71 | ID2 | na | 32767 | -0.370 | -0.4163 | Yes |
| 72 | SMAD9 | na | 32799 | -0.372 | -0.3933 | Yes |
| 73 | COMP | na | 32809 | -0.373 | -0.3696 | Yes |
| 74 | ID4 | na | 32835 | -0.375 | -0.3463 | Yes |
| 75 | TGFB1 | na | 32868 | -0.377 | -0.3230 | Yes |
| 76 | DCN | na | 32929 | -0.382 | -0.3002 | Yes |
| 77 | TGFB3 | na | 33024 | -0.389 | -0.2780 | Yes |
| 78 | IFNG | na | 33150 | -0.402 | -0.2558 | Yes |
| 79 | BMP4 | na | 33633 | -0.460 | -0.2402 | Yes |
| 80 | SMAD7 | na | 33937 | -0.511 | -0.2162 | Yes |
| 81 | TNF | na | 34328 | -0.609 | -0.1884 | Yes |
| 82 | INHBE | na | 34413 | -0.640 | -0.1498 | Yes |
| 83 | FST | na | 34624 | -0.772 | -0.1063 | Yes |
| 84 | BMP2 | na | 34656 | -0.831 | -0.0538 | Yes |
| 85 | INHBA | na | 34674 | -0.871 | 0.0016 | Yes |
Table: GSEA details [plain text format]

  

Fig 2: KEGG\_TGF\_BETA\_SIGNALING\_PATHWAY      
 Blue-Pink O' Gram in the Space of the Analyzed GeneSet

  

Fig 3: KEGG\_TGF\_BETA\_SIGNALING\_PATHWAY: Random ES distribution      
 Gene set null distribution of ES for **KEGG\_TGF\_BETA\_SIGNALING\_PATHWAY**
